# Multidisciplinary Teams for the Management of Infective Endocarditis: A Systematic Review and Meta-analysis

**DOI:** 10.1093/ofid/ofad444

**Published:** 2023-08-21

**Authors:** Anne-Sophie Roy, Hamila Hagh-Doust, Ahmed Abdul Azim, Juan Caceres, Justin T Denholm, Mei Qin (Denise) Dong, Madeline King, Christina F Yen, Todd C Lee, Emily G McDonald

**Affiliations:** Faculty of Medicine and Health Sciences, McGill University, Montreal, Canada; Faculty of Medicine and Health Sciences, McGill University, Montreal, Canada; Division of Infectious Diseases, Allergy and Immunology, Rutgers Robert Wood Johnson Medical School, New Brunswick, New Jersey, USA; Department of Internal Medicine, Michigan Medicine, Ann Arbor, Michigan, USA; Victorian Infectious Diseases Service, Royal Melbourne Hospital, Parkville, Australia; Department of Infectious Diseases, University of Melbourne at the Peter Doherty Institute for Infection and Immunity, Melbourne, Australia; Antimicrobial Stewardship Pharmacy, New York Health and Hospitals, Bellevue Hospital, New York City, New York, USA; Outpatient Antimicrobial Stewardship Clinical Pharmacy, Cooper University Healthcare, Camden, New Jersey, USA; Division of Infectious Diseases and Geographic Medicine, University of Texas Southwestern Medical Center, Dallas, Texas, USA; Clinical Practice Assessment Unit, McGill University Health Centre, Montreal, Canada; Division of Infectious Diseases, McGill University Health Centre, Montreal, Canada; Clinical Practice Assessment Unit, McGill University Health Centre, Montreal, Canada; Division of General Internal Medicine, McGill University Health Centre, Montreal, Canada

**Keywords:** endocarditis, infectious, interdisciplinary, multidisciplinary, teams

## Abstract

**Background:**

The management of infective endocarditis (IE) is complex owing to a high burden of morbidity and mortality. Recent guidelines recommend dedicated multidisciplinary teams (MDTs) for the management of IE. The aim of this systematic review and meta-analysis was to evaluate and summarize the effect of MDT management on patient outcomes.

**Methods:**

A systematic review was performed and, where feasible, results were meta-analyzed; otherwise, results were summarized narratively. Data extraction and quality assessment were performed in duplicate. Restricted maximum likelihood random effects models were used to calculate unadjusted risk ratios and 95% CIs.

**Results:**

Screening of 2343 studies based on title and abstract yielded 60 full-text reviews; 18 studies were summarized narratively, of which 15 were included in a meta-analysis of short-term mortality. Meta-analysis resulted in a risk ratio of 0.61 (95% CI, .47–.78; *I*^2^ = 62%) for mortality in favor of a dedicated MDT as compared with usual care. Length of stay was variable, with 55% (10/18) of studies reporting an increased length of stay. Most studies (16/18, 88.9%) reported a decreased time to surgery and an increased rate of surgery (13/18, 73%). No studies reported on patient-reported outcomes.

**Conclusions:**

This is the first systematic review and meta-analysis to assess the impact of MDT management on IE. The sum of evidence demonstrated a significant association between MDTs and improved short-term mortality. Further research is needed to evaluate benefits of virtual MDT care, cost-effectiveness, and the impact on patient-reported outcomes and long-term mortality.

Despite advances in diagnosis and management, infective endocarditis (IE) has an associated mortality rate exceeding 25% [1]. Notwithstanding significant medical advancements, over the past few decades the incidence and mortality of IE have paradoxically increased in many countries [2], likely due to changing epidemiology. For instance, while rates of rheumatic heart disease in industrialized countries have declined, cases in older adults have increased because of implantable cardiovascular devices and semipermanent intravenous catheters. Furthermore, the opioid crisis has resulted in an increase in IE among people who inject drugs: a population that faces challenges with respect to timely diagnosis, appropriate treatment, adherence, and adequate follow-up—all exacerbated by the high burden of stigma [3] and ongoing risk of opiate-associated death.

In addition, the management of IE has always been complex given its propensity to cause cardiac, vascular, immunologic, neurologic, and renal complications [4]. Other factors, including delays in diagnosis and/or surgical management, may further increase the risk of mortality in patients with IE [5]. The subtype of IE (native vs prosthetic valve or implantable cardiac device endocarditis) may require access to specialized imaging modalities and expert interpretation for accurate diagnosis. Finally, surgical management of endocarditis and any associated surgical complications often require specialized perioperative and surgical care.

Recognizing this interdisciplinary complexity, recent guidelines have proposed that a multidisciplinary approach be employed to the diagnosis and management of IE to achieve optimal outcomes. For example, the European Society of Cardiology’s 2015 guidelines strongly encouraged IE management by a specialized team in reference centers (implying that patients diagnosed in community hospitals or less resourced centers might need to be transferred) based on “the weight of evidence in favor of efficacy” [6]. Similarly, the 2020 American College of Cardiology/American Heart Association guidelines recommended consultation with a multispecialty heart valve team as a strong recommendation based on moderate quality evidence (nonrandomized) [7]. Whether such a consultation could be virtual was not addressed.

Indeed, several published case series and observational studies have reported improved patient outcomes with the involvement of subspecialty expertise [8–11]. The aim of this narrative review and meta-analysis was to evaluate and, when feasible, combine the totality of the evidence to determine the overall impact of multidisciplinary endocarditis teams with an emphasis on mortality.

## METHODS

### Data Sources and Search Strategy

We followed the MOOSE criteria (Meta-analysis of Observational Studies in Epidemiology) [12] and PRISMA guidelines (Preferred Reporting Items for Systematic Reviews and Meta-analyses) [13]. To identify potentially relevant studies for inclusion, we searched PubMed, CINAHL, and EMBASE for articles discussing multidisciplinary endocarditis management. Database search strategies are provided in the supplementary materials (Supplementary Tables 1 and 2). Results were extracted from database inception to 1 July 2022. Of 1889 initial results, there were 1465 nonduplicate records. Using the Rayyan platform [14], 2 independent blinded reviewers (H. H.-D. and A.-S. R.) screened articles for relevance by title and abstract. Conflicts between the reviewers were discussed and resolved through consensus.

### Study Selection

We included studies assessing the impact of a multidisciplinary management team (primary exposure) in hospitalized adult patients diagnosed with endocarditis and reporting at least 1 of the following outcomes: short-term mortality (in hospital and/or up to 30 days postdischarge—primary outcome), longer-term mortality (1 year), morbidity (renal failure, cardiac or neurologic complications, length of hospital stay, readmission), adherence to treatment, patient satisfaction, and surgical outcomes (overall rate of surgery, time to surgery). Studies limited to pediatric patients, case reports, and publications available only as poster abstracts were excluded.

### Data Extraction and Quality Assessment

Data extraction was performed in duplicate and included author, year of publication, years of data collection, location (country and type of facility), study design, population, sample size, comparator group, details of the intervention (team members, standardized protocol, and other characteristics), as well as outcomes (Table 1). Studies in the meta-analysis of short-term mortality were assessed for quality independently and in duplicate (H. H.-D. and A.-S. R.) with the Newcastle-Ottawa Scale [15]. We considered studies with 7 to 9 stars as high quality, 4 to 6 as fair, and 0 to 3 as poor.

**Table 1.** Characteristics of Studies

			Intervention			
Study	Years, Country, Facility, and Type	Population and Sample Size	Team Members	Standardized Protocol	Other Aspects	Comparator
Bain (1988) [16]	1976–1984 UK District general hospital Retrospective	Patients diagnosed with confirmed IE (clinical features suggestive of endocarditis with positive blood cultures, postmortem evidence of endocarditis, or clinical features of endocarditis with positive serologic, echocardiographic, or surgical evidence despite negative blood cultures) 75 (vs 70 in literature)	Cardiologist, specialist in ID, microbiologist, ± cardiac surgeon if necessary	Patients were referred to and managed by a preexisting MDT as soon as the diagnosis was considered.	…	Patients previously reported by Schnurr et al in whom the same diagnostic criteria were used and who were mainly treated in the same hospital before implementation of intervention (1973–1976)
Botelho-Nevers (2009) [17]	1991–2006 France Tertiary care teaching hospital Retrospective (medical records 1991–1994) then prospective (questionnaire 1994–2006)	Patients diagnosed with definite IE per modified Duke criteria. Excluding patients with IE related to gram-negative rods, fungi, or rare causes—*Legionella*, *Chlamydia*, *Brucella*, *Coxiella*, *Tropheryma* 333 episodes of IE in 321 patients (173 preintervention, 160 postintervention)	Cardiologists, cardiac surgeons, ID specialists, microbiologists	…	Follow-up: systematically seen by cardiologist and ID consultant after 1, 3, 6, and 12 mo following the end of treatment, with blood tests ± blood culture and systematic serologic testing, chest x-ray and echocardiogram	Patients before implementation of intervention (1991–2001)
Carrasco-Chinchilla (2014) [18]	1996–2011 Spain Tertiary care hospital, referral center Prospective	Patients diagnosed with LSIE per modified Duke criteria 227 (155 preintervention, 72 postintervention)	3 key domains: clinical (mainly internal medicine and ID), microbiological, and echocardiographic. An attending physician was appointed in each area	Physicians communicate by telephone if there are findings compatible with infective IE. If there is a positive diagnosis, cardiac surgery service is involved.	…	Historical cohort of patients diagnosed with left-sided IE at the same center from 1996 to 2007
Chirillo (2013) [19]	1996–2009 Italy Tertiary care hospital Prospective	Patients diagnosed with definite NVE per modified Duke criteria 292 (102 preintervention, 190 postintervention)	Cardiologist, ID specialist, microbiologist, cardiac surgeon	Standardization of blood cultures and echocardiography, initial MDT evaluation within 12 h of admission/diagnosis, decision for medical management vs early surgery	Clinical, echocardiographic, and laboratory follow-up at 1, 3, 6, 12, 24, and 36 mo after discharge	Patients before implementation of intervention (1996–2002)
Chirillo (2013) [20]	1996–2009 Italy Tertiary care hospital Prospective	Patients diagnosed with definite PVE per modified Duke criteria 99 (38 preintervention, 61 postintervention)	Cardiologist, specialist in ID, microbiologist, cardiac surgeon	All patients with suspected PVE underwent TEE, initial MDT evaluation within 12 h, with assessment of urgent surgery vs medical management. Clinical course evaluated daily and CRP, blood cultures, and plasma concentrations of antibiotics assessed every 3 d, repeat TEE, and evaluation by MDT every week.	…	Patients before implementation of intervention (1996–2002)
Diab (2021) [21]	2007–2018 Germany Tertiary care hospital Retrospective	Patients diagnosed with IE and surgically treated 630 (409 before 2015, 221 after 2015)	Cardiologists, cardiac surgeons, ID physicians, anesthesiologists, neurologists	ID consultation within 6 h, empirical antimicrobial treatment started immediately then modified after receiving blood cultures results, ID specialist determined an individualized treatment, TEE performed, consultation of MDT. After surgery, ID specialist determined antimicrobial treatment.	Expand the activities of the MDT beyond the hospital to include other referring centers and cardiologists (symposia and meetings with referring cardiologists and family doctors to increase awareness). Established an ID telephone consultation service for referring hospitals, available 24/7.	Patients before 2015 vs after January 2015 (management by the MDT increased progressively)
El-Dalati (2022) [22]	2014–2015 and 2018–2019 USA Tertiary care hospital Retrospective	Patients diagnosed with definite endocarditis per Duke criteria and at least 1 indication for surgery per the AHA guidelines 124 (68 preintervention, 56 postintervention)	Infectious diseases, cardiology, cardiac surgery, neurology, pharmacy	Cases identified through ID and cardiac surgery consult services. MDT presented IE cases at weekly conference, patient charts reviewed by ID fellow, MDT established a consensus plan that was documented in the medical record and communicated to the primary medical team.	…	Patients before implementation of intervention
Elad (2022) [23]	2013–2019 Israel Tertiary care hospital, reference center Retrospective	• Patients diagnosed with definite IE per modified Duke criteria • 220 (92 preintervention, 128 postintervention)	Led by general cardiologists. Collaboration with echocardiography, cardiac imaging, nuclear medicine, ID, cardiac surgeons. Additional consultants: electrophysiology, adult congenital heart disease, nephrologists, stroke team	Each patient with suspected IE underwent routine evaluation (blood cultures and TTE ± TEE), any case requiring further evaluation was referred to the MDT. MDT discussed cases in ad hoc meeting.	All patients were followed up at the cardiology clinics 1–6 mo after discharge.	Patients before implementation of intervention
Kaura (2017) [24]	2009–2015 UK Tertiary care hospital, reference center Prospective	Patients diagnosed with definite acute IE per modified Duke criteria 196 (101 preintervention, 95 postintervention)	2 cardiologists, 1 microbiologist, 1 cardiac imaging specialist, 1 cardiac surgeon, and an IE specialist nurse coordinator	Referral pathway: initially discussed with cardiology, TTE ± TEE, also discussed with microbiology, blood cultures taken. New referrals to the MDT were discussed immediately, existing cases at once-weekly MDT meeting, and all cases reviewed on twice-weekly ward rounds and board rounds.	All patients followed up at 1, 6, and 12 mo following discharge at a clinic run by the MDT	Patients before implementation of intervention
López-Dupla (2006) [25]	1990–2004 Spain Referral teaching hospital (no cardiac surgery) Case-control	Patients >14 y old diagnosed with IE, identified or possible IE per Durack’s modified criteria. Excluding episodes associated with a pacemaker. 120 (91 preintervention, 29 postintervention)	Internal medicine, cardiology, infective diseases and microbiology. Consultant cardiac surgeon attending monthly	…	Consultant cardiac surgeon facilitating virtual communication with the heart surgery service, IE patients needing surgical treatment transferred to the referral center.	Patients before implementation of intervention
Molnar (2021) [26]	2001–2018 Romania Heart institute Retrospective	• Patients diagnosed with IE per modified Duke criteria • 306 (mortality data: preintervention has 41 and postintervention has 23)	Cardiologist, cardiac surgeon, ID, and anesthesiologist	Surgical indication for emergency or elective intervention was established by the MDT	…	Patients before implementation of intervention (2011–2014)
Paras (2021) [27]	2016–2020 USA Academic center Retrospective (review of medical records + survey)	Patients discussed at DUET team meetings who underwent cardiac surgery for DUA-IE management 46 (27 preintervention, 19 postintervention)	Core group: cardiac surgeons, ID specialists, addiction medicine specialists, and cardiologists. Routinely invite: interventional cardiologists, neurologists, neurosurgeons, nurses, pharmacists, substance use disorder recovery coaches, case managers, social workers	Engagement of the MDT as standard practice when surgery service is consulted for a patient who injects drugs. Referral includes an online reporting form that notifies the MDT that a meeting should be scheduled. Patients reviewed via 2 meeting types: scheduled monthly and additional ad hoc.	…	Patients who underwent cardiac surgery for DUA-IE in the 2 y before implementation of intervention (2016–2018)
Ray (2020) [28]	2015–2018 USA 38-bed community-based quaternary care hospital Retrospective	Patients >18 y old diagnosed with IE and ≥1 opioid-related use disorders 70 (37 preintervention, 33 postintervention)	Cardiovascular surgery, behavioral health/addiction medicine, pain medicine, pharmacy, cardiac nursing, social work	Development and utilization of targeted cardiac surgery care medication pathway. Physician order sets were updated to carry out programmed orders.	Caregiver education	Patients before implementation of intervention
Ruch (2019) [29]	2012–2017 France University hospital, referral center Prospective (postintervention) vs retrospective (preintervention) cases	Patients >18 y old diagnosed with definite IE per modified Duke criteria. Excluding patients with ventricular assist devices 391 episodes of IE in 369 patients (316 preintervention, 75 postintervention)	Cardiac surgeons, cardiologists, echocardiographers, and ID specialists	MDT meets systematically on a weekly basis to discuss all IE cases	…	Patients in the previous 5 y before implementation of intervention
Sadeghpour (2021) [30]	2007–2019 Iran Tertiary care cardiac and medical research center Retrospective (preintervention) then prospective (postintervention)	Patients >18 y old diagnosed with definite or possible IE based on modified Duke criteria. Excluding subsequent ruling out of IE diagnosis or incomplete treatment 645 (445 preintervention, 200 postintervention)	2 specialists in ID, an echocardiologist with extensive experience in the interpretation of valvular diseases, a cardiovascular surgeon, a general cardiologist, and an epidemiologist	Each patient evaluated in organized weekly or biweekly sessions	National registry of IE cases implemented at the same time as MDT	Patients before implementation of intervention (retrospective data)
Tan (2018) [31]	2013–2017 Canada Tertiary care teaching hospital Before-and-after study (retrospective before intervention, then prospective)	Patients diagnosed with definite or probable IE per Duke criteria. Retrospective: all patients who received an *ICD-10* code for IE in any of their diagnoses + review of the hospital's registry for heart valve repair and replacement surgery and the microbiology database for at least 1 positive blood culture for microorganisms that commonly cause IE. Prospective: patients identified by notification from cardiac surgery, cardiology, ID, or GIM services. 177 (97 preintervention, 80 postintervention)	Cardiac surgery, cardiology, critical care, ID	Working group member notified of IE patient admission, face-to-face or electronic case conference discussion depending on complexity of patient, consensus recommendations communicated to treating team	…	Patients before implementation of intervention (retrospectively identified via medical records)
Van Camp (2021) [32]	2014–2019 Belgium Referral hospital Prospective	Patients diagnosed with definite IE per current ESC guidelines 160 (68 preintervention, 92 postintervention)	Cardiologists from the coronary care unit, cardiologists from the imaging and critical department, cardiac surgeons, microbiologists, neurologists, radiologists, and specialists from nuclear medicine	Patients with a suspicion of IE were immediately admitted into the coronary care unit for close monitoring, MDT gathered the same day to determine initial treatment, then discussed weekly in the MDT meetings	…	Patients before implementation of intervention
van den Heuvel (2022) [33]	2017–2018 and 2019–2020 Netherlands Tertiary care hospital Retrospective	Patients >18 y old treated for IE. Excluding patients with a CIED infection 90 (45 preintervention, 45 postintervention)	Cardiologists, cardiac surgeons, ID specialists, clinical microbiologists, nuclear medicine physicians, ± anesthesiologist	All cases discussed 1/wk in the MDT	…	Patients 1 y before implementation of intervention

Abbreviations: AHA, American Heart Association; CIED, cardiac implantable electronic devices; CRP, C-reactive protein; DUA-IE, drug use–associated infective endocarditis; DUET, drug use endocarditis treatment; ESC, European Society of Cardiology; GIM, general internal medicine; ID, infectious disease; IE, infectious endocarditis; LSIE, left-sided endocarditis; MDT, multidisciplinary team; NVE, native valve endocarditis; PVE, prosthetic valve endocarditis; TEE, transesophageal echocardiography; TTE, transthoracic echocardiography.

### Data Synthesis and Analysis

We used restricted maximum likelihood random effects models to calculate meta-analytic unadjusted risk ratios and 95% CIs. Heterogeneity was assessed with the *I*^2^ statistic. A 2-sided *P* < .05 was considered statistically significant. All analyses were performed with the meta module in Stata version 16.1 (StataCorp LP).

## RESULTS

**Figure 1.**
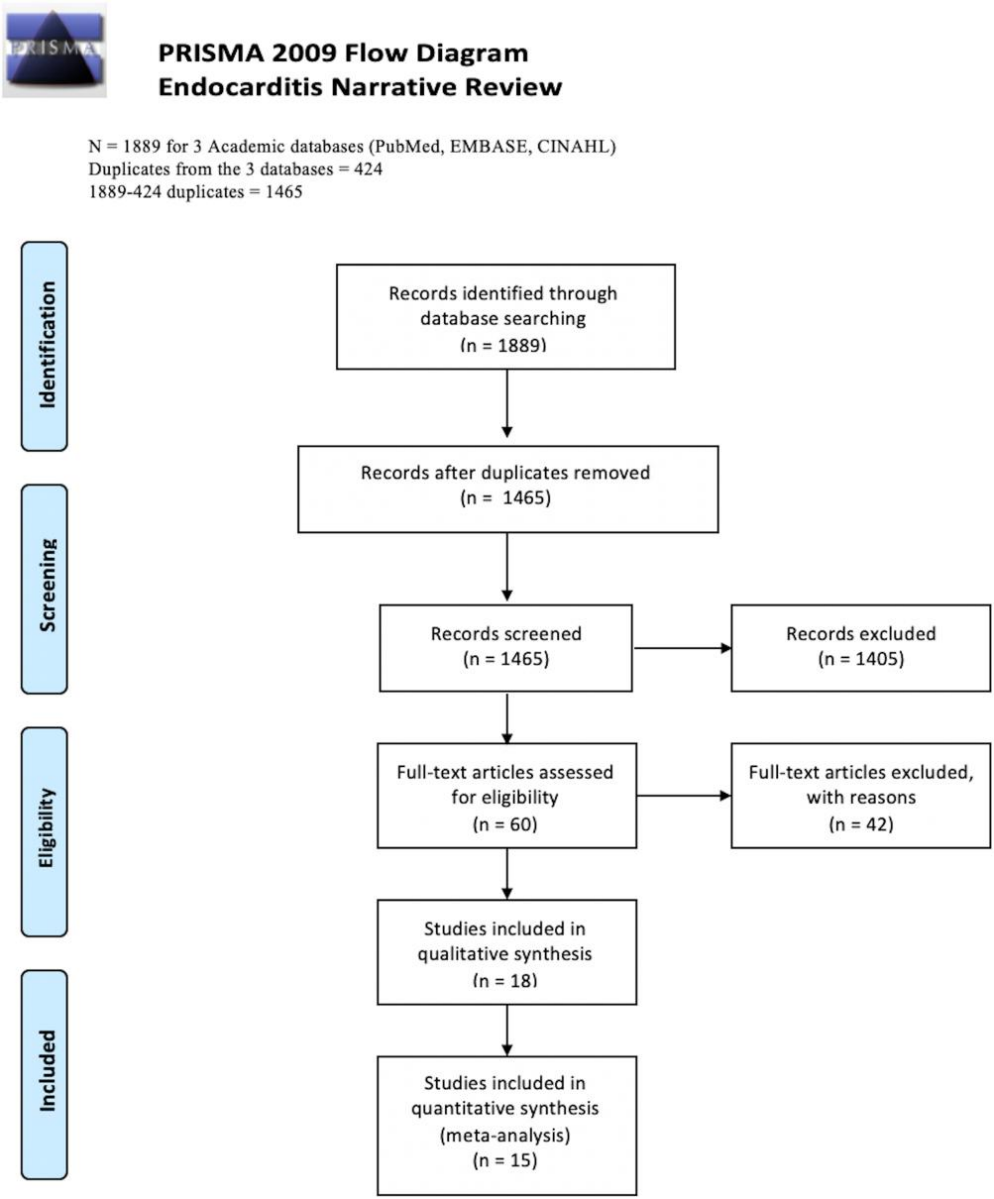
PRISMA flowchart.

We identified 1465 unique records from our preliminary search strategy. Following screening procedures, 18 were included [16–33] (Figure 1).

### Study Population

In total, 3993 episodes of IE were included: 2305 patients from preintervention/control groups, 1654 from postintervention cohorts, and 34 cases where a patient had >1 episode of IE. Sample sizes varied greatly, ranging from 6 to 645 patients. Across all studies, the mean age was 41.4 years, and 29.7% of patients were female. With respect to study type, 8 of 18 (44%) were retrospective; 5 (28%) were prospective; 4 (22%) were mixed (prospective interventional data collection with retrospective control period data); and 1 (6%) was a case-control study. All studies were conducted in single centers, most taking place at a tertiary care facility or reference center for IE; 15 studies (83%) explicitly mentioned this. Study settings were scattered across 12 countries, with all but 1 (94%) being high income. Most studies were conducted after 2010.

### Primary Outcome: In-hospital Mortality

Meta-analysis for in-hospital mortality was performed for 15 of 18 studies [16–26, 29–32]. Three studies were excluded from the meta-analysis. One study did not report any mortality data [28]. Another did not indicate in-hospital mortality [27]. One study [33] was excluded from the meta-analysis due to the implementation of an endocarditis cardiac team during the control phase (with an interventional cardiologist, an imaging cardiologist, and a cardiothoracic surgeon), which we judged too similar to the overall intervention.

**Figure 2.**
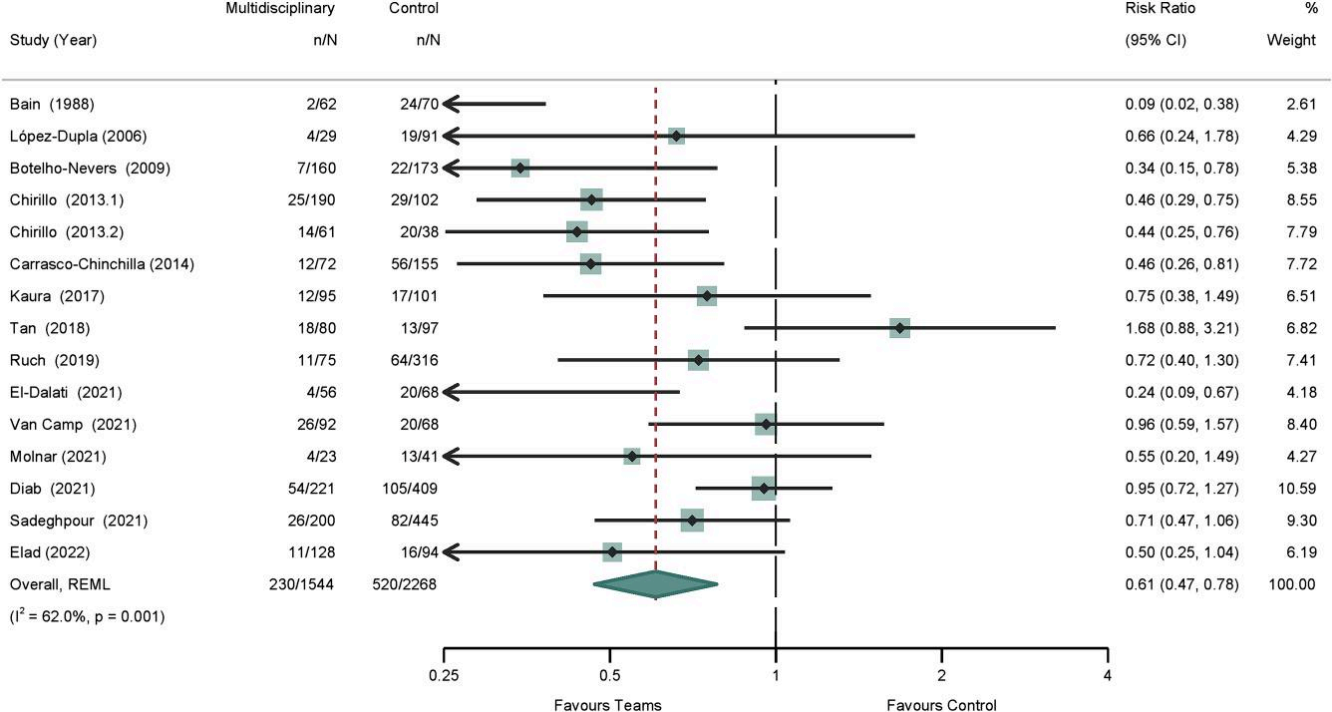
Short-term mortality of patients with infective endocarditis. REML, restricted maximum likelihood.

The meta-analysis revealed an overall risk ratio of 0.61 (95% CI, .47–.78) for patients treated with a dedicated multidisciplinary endocarditis team when compared with control groups (Figure 2). The *I*^2^ value was 62%, indicating moderate heterogeneity. Of the 15 studies assessed for quality per the Newcastle-Ottawa Scale, 12 (80%) were considered high quality and 3 (20%) fair by both reviewers (Supplementary Table 3).

### Additional Analyses to Address Confounding Factors

Though not amenable to meta-analysis, several of the 15 studies that reported on in-hospital mortality data performed additional analyses that attempted to adjust for confounding (Table 3). Most studies (11/15, 73%) performed a multivariate analysis examining factors associated with mortality. Of these, 8 (73%) demonstrated that multidisciplinary team (MDT) treatment was independently associated with reduced mortality. Three studies [17, 19, 20] included calendar year as a term in the regression model to limit confounding by other modifications in IE care that may have occurred over time. Each observed that MDT management was still associated with reduced mortality after adjusting for the calendar year. Two studies [22, 32] also performed an analysis with propensity score matching between cohorts; both revealed a reduction in mortality with MDT management.

### Descriptive Outcomes

With regard to the composition of teams, all IE teams included cardiac surgery. All but 1 (94%) [28] included cardiology and infectious diseases. Other common specialties were neurology, echocardiography/radiology, and microbiology. Most teams (10/18, 56%) met on a regular basis, which varied from weekly to monthly. Two teams (11%) met on an ad hoc basis. Six studies (33%) reported that patients received a multidisciplinary assessment but did not explicitly define any meeting frequency.

### Other Outcomes Postimplementation

Most other outcomes were reported inconsistently among studies (not amenable to meta-analysis) and so are described narratively (Table 2). No study adequately adjusted for the competing risk of death in evaluating any of the durations. Regarding length of stay, results were mixed, as was reporting (means vs medians). Eleven studies (61%) cited lengths of stay that varied from 13.5 to 42.1 days. Of the 11 studies that reported this outcome, 6 (55%) indicated an increased length of stay and 5 (45%) identified a decrease, but only 4 (36%) were statistically significant. As for surgical outcomes, time to surgery was noted in 8 studies. A majority (7/8, 88%) revealed a decrease in the time to surgery. Rate of surgery was reported in 11 studies, of which 8 (73%) noted an increased rate. Only 3 studies reported on adherence to treatment, all of which (100%) cited increased adherence to appropriate antimicrobial therapy. No studies reported on quality of life or patient satisfaction.

**Table 2.** Length of Stay and Time to Surgery for Patients With Infective Endocarditis

	Length of Stay, d			Time to Surgery, d			
Study	Preintervention	Postintervention	*P* Value	Preintervention	Postintervention	*P* Value	Definition
Bain (1988) [16]	NR			NR			
Botelho-Nevers (2009) [17]	NR			14 (0–150)^a^	11 (0–76)^a^	.35	From initiation of antibiotics
Carrasco-Chinchilla (2014) [18]	36.4^b^	37.4^b^	NR	15.1 (21.0)^b^	14.5 (20.9)^b^	.85	From admission
Chirillo (2013) [19]	NR			NR			
Chirillo (2013) [20]	NR			NR			
Diab (2021) [21]	17 (10–28)^a^	20 (14–28)^a^	.029	17 (9–37)^a^	9 (4–21)^a^	<.001	From symptoms
El-Dalati (2022) [22]	18 (19.6)^b^	24.7 (17.4)^b^	.03	14.0 (8.8)^b^	11.4 (10.1)^b^	.29	From admission
Elad (2022) [23]	27 (21.7)^b^	24.7 (17.5)^b^	.11	7.9 (8.5)^b^	10.2 (11.9)^b^	.4	Does not define time to surgery
Kaura (2017) [24]	29.2 (15.9)^b^	24.7 (17.6)^b^	.02	7.8 (7.3)^b^	5.3 (4.2)^b^	.004	From suspected IE
López-Dupla (2006) [25]	NR			NR			
Molnar (2021) [26]	NR			NR			
Paras (2021) [27]	21.8 (18.2–38.8)^a^	29.9 (17.7–38.8)^a^	.7	NR			
Ray (2020) [28]	14 (3–55)^a^	17 (2–54)^a^	.83	NR			
Ruch (2019) [29]	40.6 (22.0)^b^	31.9 (19.5)^b^	<.01	16.4 (15.0)^b^	10.3 (7.5)^b^	.049	From the definite diagnosis of IE
Sadeghpour (2021) [30]	32.85 (19.06)^b^	42.07 (17.92)^b^	.02	NR			
Tan (2018) [31]	14 (9–26)^a^	13.5 (7–21.25)^a^	.2	8 (5–14)^a^	6.5 (3.75–11.25)^a^	.43	From admission
Van Camp (2021) [32]	44.40 (32)^b^	36.1 (25.2)^b^	.0828	NR			
van den Heuvel (2022) [33]	17 (10–34)^a^	19 (11–34)^b^	.97	NR			

Data are presented as mean (SD) or median (IQR) unless noted otherwise.

Abbreviations: IE, infectious endocarditis; NR, not reported.

a Median (range).

b Mean (standard deviation).

**Table 3.** Additional Analyses Performed in Studies Examining the Effect of Multidisciplinary Team Implementation on Mortality

Study	Additional Analyses	Findings	Measure of Impact^a^	Notes
Bain (1988) [16]	None	…	…	…
Botelho-Nevers (2009) [17]	Multivariable analysis of 1-y mortality	After adjusting for changes in relevant prognostic factors and changes in baseline clinical and microbiological characteristics of patients between the periods, treatment during MDT period remained independently and strongly predictive of 1-y survival.	Adjusted HR = 0.25 (.12–.52), *P* < .001	The calendar year was added as a term in the Cox model to limit confounding by other modifications to IE care over time. The beneficial effect remained when the calendar year was added to the Cox model: adjusted HR = 0.26 (.09–.76), *P* = .01.
Carrasco-Chinchilla (2014) [18]	Univariate analysis of in-hospital mortality	The MDT strategy showed a protective role.	OR = 0.35 (.17–71.3), *P* = .003	…
	Multivariate analysis of in-hospital mortality	The MDT strategy was significantly associated with mortality regarding its protective role.	OR = 0.27 (.10–.71), *P* = .008	…
Chirillo (2013) [19]	Multivariable analysis of 3-y mortality	After adjustment for relevant prognostic factors and patient characteristics that had changed significantly between the periods, treatment during MDT period remained independently predictive of 3-y survival.	OR = 0.38 (.11–.76), *P* = .03	The beneficial effect remained when the calendar year was added to the Cox model: OR = 0.41 (.19–.88), *P* = .03.
Chirillo (2013) [20]	Multivariable analysis of 3-y mortality	After adjustment for relevant prognostic factors and patient characteristics that had significantly changed between the periods, treatment during MDT period remained independently predictive of 3-y survival.	OR = 0.55 (.22–.98), *P* = .04	The beneficial effect remained when calendar year was added to the Cox model.
Diab (2021) [21]	Multivariate binary logistic regression models of in-hospital mortality	Lack of MDT management recommendations was an independent predictor for in-hospital mortality.	Adjusted OR = 2.12 (1.27–3.53), *P* = .004	…
Elad (2022) [23]	Multivariable analysis of risk factors for long-term mortality	MDT was not associated with mortality.	HR = 1.01 (.67–1.96), *P* = .621	…
El-Dalati (2022) [22]	Multivariate binary logistic regression of in-hospital mortality	Identified the year of IE diagnosis as an independent predictor of in-hospital mortality, with patients in the post-MDT year having a lower likelihood of mortality when controlling for other variables.	Estimate = 1.70, SE = 0.547, *P* = .001	…
	Propensity score matching	Similar findings were obtained with propensity-matched outcomes between the cohorts	Overall in-hospital mortality: 5.7% post-MDT vs 34% pre-MDT	…
Kaura (2017) [24]	Multivariate analysis of the predictors of 1-y mortality	The involvement of the MDT was a significant independent predictor of improved 1-y survival in patients managed medically.	HR = 0.24 (.07–.87), *P* = .03	All demographic, clinical, and relevant outcome data that were significant in a univariate model were entered into a multivariate model
López-Dupla (2006) [25]	None	…	…	…
Molnar (2021) [26]	None	…	…	…
Ruch (2019) [29]	Multivariate analysis of in-hospital mortality	The post–MDT period was independently associated with lower in-hospital mortality.	OR = 0.45 (.20–.96), *P* < .05	Significant variables in the univariate analysis were included in the multivariate analysis
Sadeghpour (2021) [30]	None	…	…	…
Tan (2018) [31]	Multivariable logistic regression analyses of mortality 90 d after hospital discharge or a composite of mortality and new or worse complications.	After adjusting for patient age, microbiologic etiology, and heart failure at admission, the case conferencing intervention was not associated with a significant difference in mortality up to 90 d after hospital discharge or a composite of mortality and new or worse complications.	Mortality at 90 d: OR = 1.87 (.88–3.99). Composite: OR = 0.86 (.46–1.58).	Analyses limited to patients with definite IE by Duke criteria yielded results similar to the main analysis
Van Camp (2021) [32]	Cox regression models to assess independent predictors of mortality in hospital, at 6 mo, and at 1 y. Propensity score matching with a nearest neighbor	Unadjusted and adjusted mortality after propensity score matching showed no difference in in-hospital mortality, but mortality at 6 mo and 1 y tended to be lower in patients managed by the MDT.	Mortality at 6 mo: 23.5% in MDT cohort vs 36.8% in controls, *P* = .0926. At 1 y: 26.5% in MDT cohort vs 41.2% in controls, *P* = .0699	…

Abbreviations: IE, infective endocarditis; HR, hazard ratio; MDT, multidisciplinary team; OR, odds ratio.

a Data in parentheses indicated 95% CI.

## DISCUSSION

To our knowledge, this is the first systematic review and meta-analysis to assess the impact of multidisciplinary endocarditis team management. While limited to single-center observational studies, the data suggest that IE team implementation is associated with decreased short-term mortality, which, if correct, is clinically important. We agree with authors who have previously hypothesized that the mortality reduction achieved by MDTs is likely multifactorial. Potential mechanisms were as follows: earlier detection, diagnosis (including reduced time to echocardiography), and initiation of treatment; decreased time to surgery; earlier and more frequent assessments by infectious diseases consultants; improved adherence; and more appropriate treatments (antimicrobial agent, dose, duration).

We noted variability in the length of stay across studies pre- and postintervention. This is consistent with Bikdeli et al [34], who examined trends in length of stay in a large cohort of hospitalized patients with IE and found it to be highly variable. When length of stay is evaluated, adjustment for the competing risk of death will be very important. Multidisciplinary teams could increase length of stay for various reasons, such as delayed discharge due to more investigations, a higher rate of surgery, or, paradoxically, higher survival. Yet, a decreased length of stay might be possible if the MDT model renders the process of hospital discharge more efficient. Consequently, length of stay may not be a suitable outcome to demonstrate the success of IE teams.

Increased rates of surgery and decreased time to surgery were noted in many studies reporting these outcomes. This is in line with Regunath et al [35], where they deployed a series of quality improvement tools to improve multidisciplinary IE care and identified that the decision to operate was a leverage point to improve care. Strategies include early identification of patients at high risk to quickly escalate the evaluation for surgical management. As “any solution for this leverage point will mandate collaboration of all of the essential subspecialty services” [35], one can hypothesize that reduced time to surgery could be achieved with earlier identification of urgent cases and a facilitated, prompt, direct communication among involved experts. Nonetheless, since not all studies reported on this outcome, it is subject to a reporting bias.

Our review had strengths, such as its systematic approach and large number of identified patients. It also aligns with and provides stronger supporting data for the specialty guidelines [6], which suggest that patients with IE should be managed by specialized endocarditis teams that meet on a regular basis and select therapies based on the best available evidence.

There are limitations to this review worth discussing in some detail, many of which are inherent to the design and outcome measurements in the studies. For instance, most studies were designed as pre- and postintervention groups, which are not ideal to meta-analyze because they are not contemporaneous and thus are subject to bias due to temporal changes in outcomes that confound the relationship between outcome and intervention. Mortality reductions might therefore reflect general improvements in medical knowledge and care arising during the elapsed time between pre- and postintervention measurements rather than the advantage of the MDT management itself. Along these lines, studies performed prior to 2014 contributed substantially to the observed overall reduction in mortality. However, it should be noted that mortality from IE has been increasing over time [2]. As such, improvements seen postintervention would go against the general time trend of an observed increase in mortality. As mentioned, while several studies did perform multivariate analyses to adjust for potential confounders, only 3 [17, 19, 20] included calendar year as a term in their models to limit this form of bias. In these 3 studies, MDT management remained beneficial.

There are other limitations worth mentioning. First, the observational nature of the studies risks residual confounding, confounding by indication, immortal time, temporal trends, selection bias, reporting bias, and other biases. As many of these centers were referral centers, it is also impossible to know whether there were systemic biases in who was even evaluated and accepted for transfer. Second, even the primary mortality outcome was subject to moderate heterogeneity. This could be due to several factors, such as the lack of a standardized intervention design and the diversity across the studies in context, location, and sample size. Third, meta-analysis of other outcomes was not feasible given the lack of consistent reporting. Whereas mortality from IE is generally affected by many factors—an important one being the timing of surgery among patients with surgical indications—most studies did not report this outcome. Among the few studies that reported time to surgery, definitions were variable, making it impossible to meta-analyze the results. Fourth, we included all types of IE cases, but people who inject drugs and patients with cardiac-implanted devices have several specific diagnosis and management challenges [36]. Fifth, studies were mostly conducted in high-income countries; therefore, the findings may not be generalizable to less-resourced settings. Studies did not evaluate cost-effectiveness; many were conducted in cardiac referral centers; and there was little discussion surrounding how to operationalize patient transfers or how to ensure equitable access to the required expertise in remote or underserved populations. Sixth, the overall proportion of female patients in our review was lower than the prevalence of IE among females in the literature, so this population was underrepresented in the studies [37]. Last, apart from adherence to treatment, none of the studies examined the impact on any patient-reported outcomes.

While MDTs seem reasonable on face value and are supportable by this analysis, the answer to many of the methodological limitations of the observational evidence will be to perform randomized controlled trials. MDTs represent a substantial investment of health care resources and may require an increase in patient transfers; therefore, it is reasonable to conclusively demonstrate value to justify those investments. Additionally, publishing guidance on a standardized framework for MDT management could be helpful, especially if such guidance were to include meaningful outcome definitions to be used in future observational, quasi-experimental, and randomized studies.

## CONCLUSION

This systematic review and meta-analysis demonstrated a significant association between team-based management and reduced short-term mortality. While this supports the current guideline recommendations, the resources required for universal deployment are substantial, and deployment will not be immediate or simultaneous. As such, a cluster randomized or stepped-wedge controlled trial could serve as a means of scalable deployment and an evidence-based approach to measuring the impact on important outcomes.

## Supplementary Material

ofad444_Supplementary_Data

## Supplementary Data

Supplementary materials are available at *Open Forum Infectious Diseases* online. Consisting of data provided by the authors to benefit the reader, the posted materials are not copyedited and are the sole responsibility of the authors, so questions or comments should be addressed to the corresponding author.

## Notes

***Acknowledgments.*** E. G. M. is a board member of WikiGuidelines, and T. C. L. sits on the steering committee of WikiGuidelines. WikiGuidelines is a not-for-profit company in the United States that crafts guidelines for the management of infectious diseases.

***Author contributions.*** Conceptualization: all authors. Methodology: E. G. M. Validation: T. C. L. Formal analysis: E. G. M. and T. C. L. Investigation: all authors. Data curation: A.-S. R., H. H.-D. Writing–original draft: A.-S. R., H. H.-D., and E. G. M. Writing–review and editing: all authors. Visualization: E. G. M., A.-S. R., H. H.-D., T. C. L.
