# Supplementary material for: Multidisciplinary Teams for the Management of Infective Endocarditis: A Systematic Review and Meta-analysis
Source: Open Forum Infect Dis. 2023 Aug 21;10(9):ofad444. doi: 10.1093/ofid/ofad444 (PMC10478154; doi:10.1093/ofid/ofad444)
Supplement: ofad444_Supplementary_Data [file ofad444_supplementary_data.docx]

Supplementary Table 1: Database Search Strategy

| Database | Search terms | Number of results |
| --- | --- | --- |
| Pubmed | ((endocarditis [ti]) OR ("Endocarditis"[Mesh])) AND (((multidisciplinary [tiab]) OR ("Patient Care Management"[Mesh])) OR (team [tiab])) | 714 |
| CINAHL | (TI endocarditis OR MH "Endocarditis") AND (TI team OR TI multidisciplinary OR MH "Multidisciplinary Care Team") | 38 |
| EMBASE | (exp endocarditis or bacterial endocarditis or endocarditis.ab, ti) AND (exp multidisplinary team or exp collaborative care team or multisciplinary.ab,ti) | 727 |

Supplementary Table 2: Article Inclusion & Exclusion criteria

| Criteria | Inclusion | Exclusion |
| --- | --- | --- |
| Type of study | - RCTs | - Case reports - Expert opinion - Editorials |
| Adults with infective endocarditis | Population studied is :   - >18 y/o - Hospitalized patients - Confirmed diagnosis of IE (either native or prosthetic valve) | Population is :   - Patients <18 y/o - Outpatients - No confirmed diagnosis IE |
| Multidisciplinary team | - Discussing a preexisting endocarditis team including members from   - Cardiology   - Infectious Diseases   - Surgery | - Not discussing multidisciplinarity - Not discussing a standardized care protocol for endocarditis |
| Outcomes | - Clinical cure - Length of hospital stay - Mortality   - In-hospital   - 1-year - Morbidity - Compliance to treatment - Surgery   - Overall rate   - Time to surgery | - Does not include one of the above outcomes |
| Comparator | - Comparing to care with no preexisting dedicated endocarditis team | - Other type of comparison group - No comparison group |

Supplementary Table 3: Newcastle-Ottawa Scale Quality Assessment

| Authors | Selection | | | | Comparability | | Outcome | | | Total points (out of 9) | Quality of study |
| --- | --- | --- | --- | --- | --- | --- | --- | --- | --- | --- | --- |
|  | Representativeness | Selection of non-exposed | Ascertainment of exposure | Demonstration that outcome was not present at start | Controls for comorbidities | Controls for age | Assessment | Follow-up long enough | Adequacy of follow-up |  |  |
| Bain (1988) | 0 | 1 | 0 | 1 | 0 | 1 | 0 | 1 | 0 | 4 | Fair |
|  | 1 | 1 | 0 | 1 | 0 | 1 | 0 | 1 | 0 | 5 |  |
| Botelho-Nevers (2009) | 1 | 1 | 1 | 1 | 1 | 1 | 1 | 1 | 1 | 9 | High |
|  | 1 | 1 | 1 | 1 | 1 | 1 | 1 | 1 | 1 | 9 |  |
| Carrasco-Chinchilla (2014) | 1 | 1 | 1 | 1 | 1 | 1 | 0 | 1 | 0 | 7 | High |
|  | 1 | 1 | 1 | 1 | 1 | 1 | 0 | 1 | 1 | 8 |  |
| Chirillo (2013a) | 1 | 1 | 1 | 1 | 1 | 1 | 1 | 1 | 1 | 9 | High |
|  | 1 | 1 | 1 | 1 | 1 | 1 | 1 | 1 | 1 | 9 |  |
| Chirillo (2013b) | 1 | 1 | 1 | 1 | 1 | 1 | 1 | 1 | 1 | 9 | High |
|  | 1 | 1 | 1 | 1 | 1 | 1 | 1 | 1 | 1 | 9 |  |
| Diab (2021) | 1 | 1 | 1 | 1 | 0 | 0 | 1 | 1 | 1 | 7 | High |
|  | 1 | 1 | 1 | 1 | 0 | 0 | 1 | 1 | 1 | 7 |  |
| El-Dalati (2021) | 1 | 1 | 1 | 1 | 1 | 1 | 1 | 1 | 0 | 8 | High |
|  | 1 | 1 | 1 | 1 | 1 | 1 | 1 | 1 | 1 | 9 |  |
| Elad (2022) | 1 | 1 | 1 | 1 | 1 | 1 | 1 | 1 | 1 | 9 | High |
|  | 1 | 1 | 1 | 1 | 1 | 1 | 1 | 1 | 1 | 9 |  |
| Kaura (2017) | 1 | 1 | 1 | 1 | 1 | 1 | 1 | 1 | 1 | 9 | High |
|  | 1 | 1 | 1 | 1 | 1 | 1 | 1 | 1 | 1 | 9 |  |
| López-Dupla (2006) | 1 | 1 | 0 | 1 | 1 | 1 | 0 | 1 | 0 | 6 | Fair |
|  | 1 | 1 | 0 | 1 | 1 | 1 | 0 | 1 | 0 | 6 |  |
| Molnar (2021) | 1 | 1 | 1 | 1 | 0 | 0 | 1 | 1 | 1 | 7 | High |
|  | 1 | 1 | 1 | 1 | 0 | 0 | 1 | 1 | 1 | 7 |  |
| Ruch (2019) | 1 | 1 | 1 | 1 | 1 | 1 | 1 | 1 | 0 | 8 | High |
|  | 1 | 1 | 1 | 1 | 1 | 1 | 1 | 1 | 1 | 9 |  |
| Sadeghpour (2021) | 1 | 1 | 0 | 1 | 0 | 0 | 0 | 1 | 0 | 4 | Fair |
|  | 1 | 1 | 0 | 1 | 0 | 0 | 0 | 1 | 1 | 5 |  |
| Tan (2018) | 1 | 1 | 1 | 1 | 1 | 1 | 1 | 1 | 0 | 8 | High |
|  | 1 | 1 | 1 | 1 | 1 | 1 | 1 | 1 | 1 | 9 |  |
| Van Camp (2021) | 1 | 1 | 1 | 1 | 1 | 1 | 1 | 1 | 1 | 9 | High |
|  | 1 | 1 | 1 | 1 | 1 | 1 | 1 | 1 | 1 | 9 |  |

First row (grey): reviewer 1 (ASR); second row (white): reviewer 2 (HHD)
